# Unhealthy Food Choices among Healthcare Shift Workers: A Cross-Sectional Study

**DOI:** 10.3390/nu14204327

**Published:** 2022-10-16

**Authors:** Anna Wolska, Beata Stasiewicz, Karolina Kaźmierczak-Siedlecka, Maciej Ziętek, Joanna Solek-Pastuszka, Arleta Drozd, Joanna Palma, Ewa Stachowska

**Affiliations:** 1 Department of Human Nutrition and Metabolomics, Pomeranian Medical University, 70-204 Szczecin, Poland; 2 Department of Human Nutrition, Faculty of Food Sciences, University of Warmia and Mazury in Olsztyn, Sloneczna 45F, 10-718 Olsztyn, Poland; 3 Department of Surgical Oncology, Faculty of Medicine, Medical University of Gdańsk, Smoluchowskiego 18, 80-214 Gdańsk, Poland; 4 Department of Perinatology, Obstetrics and Gynecology, Pomeranian Medical University, 70-204 Szczecin, Poland; 5 Department of Anesthesiology and Intensive Therapy, Pomeranian Medical University, 70-204 Szczecin, Poland; 6 Department of Biochemical Science, Pomeranian Medical University in Szczecin, 71-460 Szczecin, Poland

**Keywords:** dietary patterns, Mediterranean diet, fat intake, dietary habits, healthcare, shift workers

## Abstract

Shift healthcare workers are a group particularly exposed to an increased risk of poor eating habits and are affected by many diseases. The aim of the study was to evaluate the dietary patterns (DPs), including the Polish-adapted Mediterranean Diet (Polish-aMED^®^) score, and dietary fat intake in association with the shift work of healthcare workers. This cross-sectional study involved 445 healthcare workers from the West Pomeranian in Poland. Dietary data were collected using an FFQ-6^®^. A posteriori DPs were derived with a Principal Component Analysis (PCA). The Polish-aMED^®^ score and the individual’s percentage of energy from dietary fat (Pfat) were calculated. Healthcare shift work compared to the daily work was associated with approximately 2-times higher odds of adherence to the ‘Meat/fats/alcohol/fish’ DP in the upper tertile (OR: 2.38; 95% Cl: 1.27–4.47; *p* < 0.01) and higher Pfat >35% of total energy intake (OR: 1.73; 95% Cl: 1.06–2.83; *p* < 0.05). Healthcare shift work compared to the daily work was associated with approximately 50% lower odds of adherence to the ‘Pro-healthy’ DP in the middle tertile (OR: 0.48; 95% Cl: 0.26–0.89; *p* < 0.05) and a higher level of the Polish-aMED^®^ score (OR: 0.57; 95% Cl: 0.33–0.98; *p* < 0.05), as well as lower odds of the constants of mealtime (OR: 0.54; 95% Cl: 0.33–0.89; *p* < 0.05). The obtained findings highlight the unhealthy food choices among shift healthcare workers. Thus, to avoid the negative health consequences, there is a need for nutritional education for healthcare workers, especially those working shifts.

## 1. Introduction

The definition of the International Labour Organization describes shift work as an organization of time in which persons succeed one another in the same position. Shift work is characterized by varying shift lengths, which can be anywhere from 12 to 24 h. Notably, in 2020, shift work was performed by approximately 15–20% of employees in Europe, 20% in the United States of America, 6–32% in Asia, as well as 8% in Poland [1]. The healthcare sector is one of the professional groups that perform shift work. The medical professions are represented by paramedics, nurses, and doctors. Paramedics are employed full-time in one, two, or even three facilities, such as in emergency and private company transporting patients. They work on a contract of mandate, a contract for specific work, or a contract or a second employment contract for 1/4, ½, or even more time [2,3].

The work system has an impact on both lifestyle and health [4,5]. Notably, the possible negative consequences of working at night include dysregulation of the phases of physiological rhythms and atypical times of meals. It contributes to occurrence of problems with digestion and consequently leads to the development of digestive system disorders, sleep disorders, neuropsychic disorders, increased risk of obesity, and impaired glucose tolerance [4,5,6]. Additionally, night work negatively affects blood pressure values and thus increases the risk of cardiovascular disease [4,5,6,7]. A meta-analysis of 57 articles has shown that there is a link between shift work, especially night work, as well as seniority and the occurrence of particular types of cancer, such as colorectal, endometrial, and prostate cancer [8].

Eating meals at night may negatively affect metabolism and the absorption of nutrients as well as medications that are taken at that time [9,10]. It should be emphasized that shift workers often do not have time to eat regular and well-balanced meals that could meet their both energy and nutritional requirements. Shift workers often consume ready-to-eat and processed products/melas with low nutritional value as well as a high fat content. Thus, they often consume food that includes a high content of energy [11]. It was noted that the frequent use of processed ready-to-eat meals by night shift workers causes them to consume relatively more energy than workers who work at day [12,13]. Shift health workers often consume sandwiches, cakes, potato chips, and biscuits. In many studies, it was observed that night shift workers consume a higher amount of fried foods, which include saturated fatty acids, compared to morning and afternoon shift workers [13]. Moreover, night shift workers eat a lower amount of fruit and vegetables [14,15,16]. In another study it was shown that insufficient fruit consumption by resident doctors was positively correlated with an increase in the number of overtime hours [17].

Currently, there is no standard method that allows for diet assessment. Additionally, many approaches are now used to study diet. Characterizing eating patterns (dietary patterns, DPs) is the most common approach used to evaluating a diet [18,19]. The two most common approaches include a priori analysis, which is based on current knowledge and evidence-based relationships between diet and health, for instance the Mediterranean Diet Index [20], and a posteriori analysis, which is a research approach based on dietary data collected for the studied sample [14]. Both approaches are used in assessing the relationship diet and the health/risk of disease [21,22,23]. There is still limited research that assesses how shift work affects the eating habits and patterns of nutrition, especially among healthcare professionals. During last 10 years, only 3 papers that discussed the link between shift work and nutrition patterns have been published [24,25,26].

The aim of the study was to evaluate dietary patterns (DPs), including the Polish-adapted Mediterranean Diet (Polish-aMED^®^) score, and dietary fat intake in associations with the shift work in healthcare workers. The selected aspects of dietary habits, including regular mealtime consumption by the mode of the work among healthcare workers, were also examined.

## 2. Materials and Methods

### 2.1. Ethical Approval

The study was approved by the Bioethics Committee of the Pomeranian Medical University (consent no. KB-0012/144/18). All procedures were carried out according to the Helsinki Declaration, and the study protocol has been approved by the Bioethics Committee. All test results were confidential.

### 2.2. Study Design and Sample Characteristics

The current study was started in November 2019 and completed in April 2021. Initially, it was planned to include only doctors and nurses working in shifts (12 h/12 h or 24 h/24 h) in hospitals in Szczecin (West Pomeranian Voivodeship, Poland). Due to the SARS-CoV2 virus pandemic and its related restriction about entering the wards, data was collected online, and the study group was expanded to include all healthcare workers (i.e., doctors, nurses, paramedics) working in various shift systems (day, 12 h shift/12 h, 24 h shift/24 h, night shift). In total, this study included 445 healthcare workers—doctors and nurses working in 3 hospital departments: gynecology and obstetrics, cardiology and psychiatry, including 193 shift workers and 252 day care workers.

### 2.3. Dietary Patterns Identification

Dietary data were obtained using a validated and self-administered version of the 62-items Food Frequency Questionnaire (FFQ-6^®^) [19]. The wide scope of application of the FFQ-6^®^ questionnaire has been confirmed by its use, among others, in a pilot, controlled, and randomized study in children with celiac disease on a gluten-free diet [27], also in patients with non-alcoholic fatty liver disease [21] and among hemodialysis patients [28].

Data about the frequency of consumption of 62 food groups at least 12 months prior to participation in the study (categories) were obtained. The frequency consumption was expressed as times/day after assigning the values for categories of frequency consumption as follows: ‘never or almost never’ = 0; ‘once a month or less’ = 0.025; ‘several times a month’ = 0.1; ‘several times a week’ = 0.571; ‘daily’ = 1; ‘several times a day’ = 2. The consumption frequency of some food groups was summed up to form 23 food groups. The full description of 23 food groups aggregated was shown in Supplementary Table S1. Next, data on the frequency of consumption of 23 food groups expressed in times/day (input variables) was standardized and included in the Principal Component Analysis (PCA) with varimax rotation to identify dietary patterns (DPs). The main criteria for the PCA-derived DPs identification were the plot of eigenvalues and the total variance explained. DPs were labelled according to the main components with the value of factor loadings > |0.30| [22]. For further analysis, tertile intervals of each DP were calculated. The characteristics of the DPs were given in the Results section.

### 2.4. Polish-Adapted Mediterranean Diet Score

The Polish-adapted Mediterranean Diet (Polish-aMED^®^) score is a Polish version of the Mediterranean diet score developed by Krusinska et al. [21]. The Polish-aMED^®^ score was calculated based on the qualitative data of the frequency of consumption (times/day) of eight food items: vegetables, fruit, whole grains, fish, legumes, nuts and seeds, processed meat, and the ratio of vegetable oils to animal fat. All dietary data were obtained using the FFQ-6^®^. The difference with the original version of the Polish-aMED^®^ Score was that the group “processed meat” was included instead of the combined group “red and processed meat” due to the lack of data on red meat consumption [21]. For each of the subjects, 1 or 0 points were given for individual food items depending on the frequency of consumption. The cut-off points were medians of the frequency of consumption for the initial control sample of the case-control study conducted by Krusinska et al. [21]. The Polish-aMED^®^ score was calculated based on the sum of points assigned to the individual items. Details of the Polish-aMED^®^ score calculation are shown in Supplementary Table S2. The adherence to the Polish-aMED^®^ score was expressed in a range from 0 to 8 points and was considered at two levels: lower (0–4 points), and higher (5–8 points).

### 2.5. Dietary Fat Intake Assessment

The Quick Food Scan of the National Cancer Institute and the Percentage Energy from Fat Screener scoring procedures were used to estimate an individual’s percentage energy from dietary fat (Pfat) [23,24]. The participants were asked about the frequency of consumption of food items during last 12 months. The frequency of consumption was calculated (8 categories to select) into the number of times consumed per day as follows: ‘never’ = 0; ‘less than once a month’ = 0.018; ‘1–3 times per month’ = 0.066; ‘1–2 times per week’ = 0.214; ‘3–4 times per week’ = 0.499; ‘5–6 times per week’ = 0.784; ‘1 time per day’ = 1; ‘2 or more times per day’ = 2. The median age- and gender-specific portion sizes for each food item were multiplied by the frequency of consumption expressed in times/day.

Total fat (Tfat) was calculated as the sum of the frequency of consumption of the margarine, butter, or oil added to foods (on bread, rolls, pancakes; vegetables including potatoes; and rice or pasta). Next, the regular fat (Rfat) was calculated as a product of the Tfat and the values assigned to the frequency of reduced-fat margarine use, as follows: ‘did not use or almost never’ = Tfat *1; ‘about 1/4 of the time’ = Tfat *0.75; ‘about 1/2 of the time’ = Tfat *0.50; ‘about 3/4 of the time’ = Tfat *0.25; ‘almost always or always’ = Tfat *0. The individual’s Pfat was estimated by applying gender-specific regression coefficients to each of the 12 food items (b1–b12) and Rfat (b13) in the following algorithm [23,24]:

(1) Percentage of energy from dietary fat = intercept + (b1*cereal) + (b2*skim milk) + (b3*eggs) + (b4*bacon) + (b5*citrus juice) + (b6*fruit) + (b7*hot dogs) + (b8*cheese) + (b9*fries) + (b10*mayonnaise) + (b11*salad dressings) + (b12*rice) + (b13*regular fat)

For the Percentage of Energy from Fat Screener scoring procedures, the US Department of Agriculture 1994–1996 was the source of data for portion sizes and regression coefficients (Continuing Survey of Food Intakes by Individuals CSFII) [24,25].

### 2.6. Confounders

The socioeconomic status (SES) was calculated on the basis of three components: place of residence, declared economic situation of the household and the level of education (Table 1). For each respondent, the sum of the numerical values assigned to individual categories of SES components was calculated. In the abovementioned way, the SES index was calculated (which could be in a range from 3 to 13 points). Depending on the value of SES index, there were 3 selected categories SES: low (3–6 points), average (7–10 points), and high (11–13 points).

The Godin-Shephard Leisure-Time Physical Activity Questionnaire (GLTEQ) was used for the physical activity assessment [26]. Respondents were asked how many times on average they exercise for more than 15 min during their free time in a typical 7-day period (week). There were three categories of exercise: strenuous (heart beats rapidly, e.g., running, jogging, football, vigorous swimming, vigorous long-distance bicycling), moderate (not exhausting, e.g., fast walking, tennis, easy bicycling, easy swimming), and mild activities (minimal effort, e.g., yoga, easy walking, bowling, fishing from a riverbank) (Godin et al. 2011). Then, the total weekly leisure activity score was calculated as a sum of the products of strenuous, moderate, and mild activities expressed in the number of times/week, and the metabolic equivalent of task (MET) values corresponding to these categories: nine, five, and three, respectively, as shown in the following formula:

(2) Weekly leisure-time activity score = (9*Strenuous) + (5*Moderate) + (3*Mild)

In summary, in reference to the score in units obtained using only moderate and strenuous physical activities, respondents were categorized as: active (24 units or more), moderately active (14–23 units), and insufficiently active (less than 14 units) [26].

Besides socioeconomic status index (SESI) and physical activity (GLTEQ summary in points), the present analyses were taking into account a set of many potential confounders: age (years), gender (men, women), BMI (kg/m^2^), current smoking status (non-smoker, smoker), alcohol drinking within the last 12 months (no, yes), chronic diseases (no, yes), taking medication > 1 year (no, yes), and vitamin/mineral supplements use within the last 12 months (no, yes).

### 2.7. Statistical Analysis

All continuous data, including food frequency consumption (times/day), Polish-aMED^®^ score (points), total and regular fat intake (g), and the percentage energy from dietary fat (%) were expressed in means and standard deviations (SD). For categorical data, involving tertiles intervals or levels of DPs or Pfat, the percentage distributions were calculated. Differences in baseline sample characteristics were verified with a Kruskal–Wallis test (continuous data) or Pearson Chi2 test (categorical data). The Pearson’s correlation coefficients were calculated for 23 food items in the Polish-aMED^®^ score (Armitage et al. 2001). The percentage distribution of healthcare workers was compared in tertiles or levels of DPs using the Pearson Chi2 test with Yates’ correction as necessary. The logistic regression analysis was used to assess the associations of the adherence to the dietary patterns, percentage of energy from dietary fat, and constants of mealtime by mode of work among healthcare workers. The odds ratios (ORs) and 95% confidence intervals (95% CI) were calculated. The references (OR = 1.00) were the healthcare workers working daily, the bottom tertile or lowest level of each DP, and no constants of mealtime. Two models were created: an unadjusted model, and a model adjusted for the potential confounders mentioned above. The level of significance of the odds ratio was verified with Wald’s test (Armitage et al. 2001). The statistical analysis was performed using STATISTICA software (version 13.0 PL; StatSoft Inc., Tulsa, OK, USA; StatSoft, Krakow, Poland). A *p*-value < 0.05 was considered statistically significant.

## 3. Results

### 3.1. Baseline Sample Characteristics

This study included 445 healthcare workers, including 252 daytime workers and 193 shift workers 18–74 years old. Most of the respondents came from large cities with more than 200,000 inhabitants (52.1%) and had higher education (92.8%). Shift workers compared to day workers were younger (34.0 vs. 36.4 years; *p* = 0.0188), characterized by a lower socio-economic status index (11.2 vs. 11.6 points, *p* = 0.0045), and they significantly less frequently declared taking medications for more than one year (25.9 vs. 35.7%; *p* = 0.0272) and the use of vitamin and mineral supplements (65.3 vs. 75.0%; *p* = 0.0255). There were no significant associations in place of residence, educational level, BMI, some of health and lifestyle data such as physical activity, alcohol drinking, smoking status, or occurrence of chronic diseases depending on lifestyle of healthcare workers. The characteristics of the participants are presented in Table 2.

### 3.2. Dietary Patterns

Three totally different PCA-derived DPs were identified. The total share in explaining the variance was 38.5%. The ‘Lacto-ovo-vegetarian’ DP was characterized by a lesser frequency of consumption of snacks, sugar and sweetened foods, refined grains, and breakfast cereals, and a higher frequency of consumption of vegetables, fruits, whole grains, legumes, nuts, and seeds than the ‘Sweet-salty-snack-dairy’ DP (Table 3 and Supplementary Tables S3 and S4). The ‘Sweet-salty-snack-dairy’ DP was the opposite of the ‘Lacto-ovo-vegetarian’ pattern, i.e., it involves more frequent consumption of sweetened milk drinks/flavored cheese, salty snacks, sugar/honey/sweets, refined grains, breakfast cereals, and sweetened beverages, and much less frequent consumption of vegetables, fruits, whole grains, legumes, nuts, and seeds (Tables S3 and S4). The pattern labeled ‘Meat-fats-alcohol-fish’ exceeds the other two patterns not only for fats but also for processed meats, white meat, alcohol, and fish (Tables S3 and S4).

The a priori Polish-aMED^®^ score was positively correlated with the frequency of consumption of vegetables, fruits, whole grains, legumes, nuts and seeds, fish, and vegetable oils including olive oil, and negatively correlated with the frequency of consumption of processed meats (Table 3). The frequency of consumption of food groups and the sample characteristics by tertiles of DPs are shown in Supplementary Tables S3 and S4, respectively.

### 3.3. Food Choices and Fat Intake: Associations with Shift Work

Compared to the daily healthcare workers, significantly more shift workers were in the upper tertile of the ‘Meat-fats-alcohol-fish’ DP (42.0 vs. 27.0%), as well as having a higher total fat intake (4.1 vs. 2.9 g), and dietary fat intake of total energy intake >35% (50.3 vs. 36.1%; Table 4). On the other hand, compared to the daily workers, the number of shift workers was lower in the higher level of the Polish-aMED^®^ score (26.4% vs. 38.5%) and lower in the middle tertile of the ‘Lacto-ovo-vegetarian’ DP (26.4% vs. 38.9%). Shift workers compared to daily workers significantly less frequently declared using a special diet or restrictions in food consumption (34.2 vs. 45.8%), including dairy (21.8 vs. 34.7), fruits (5.7 vs. 12.7 %), potatoes and cereals (21.8 vs. 36.1%), or meat and meat products (33.2 vs. 50.4%), and less frequently declared constants of mealtime (44.0 vs. 62.7%). There were no significant differences in the number of shifts and daily workers within the tertiles of the ‘Sweet-salty-snack-dairy’ DP (Table 4). The mean consumption of food groups by mode of work among healthcare workers is shown in Supplementary Table S5. Associations between dietary patterns and energy from dietary fat are presented in Supplementary Table S6.

**Figure 1:**
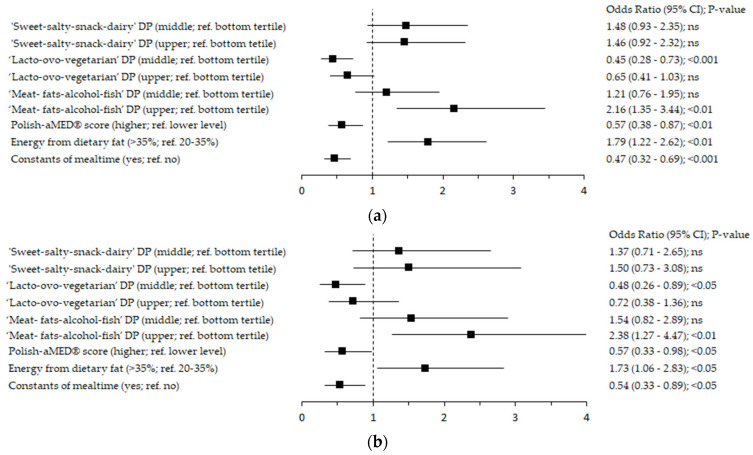
Forest plots of the adherence to the dietary patterns, percentage energy from dietary fat, and constants of mealtime among healthcare workers working in shift mode (ref. daily mode) (n = 445): (**a**) crude model; (**b**) model adjusted for: age (years), gender (men, women), socioeconomic status index (SESI, points), BMI (kg/m^2^), physical activity (GLTEQ summary, points), current smoking status (non-smoker, smoker), alcohol drinking within the last 12 months (no, yes), chronic diseases (no, yes), taking medication >1 year (no, yes), and vitamin/mineral supplements use within the last 12 months (no, yes). DP—dietary pattern; Polish-aMED^®^—Polish-adapted Mediterranean Diet (range of points: 0–8); ref.—referent, the reference categories were the bottom tertile/lower level of dietary patterns and the lower level of the percentage of energy from dietary fat; 95% CI—95% confidence interval; *p*-value—the level of significance was assessed by Wald’s test.

Healthcare shift work compared to the daily work was associated with an approximately 2-times higher odds of the adherence to the ‘Meat-fats-alcohol-fish’ DP in the upper tertile (OR: 2.38; 95% confidence interval (95% Cl): 1.27–4.47; *p* < 0.01; reference: bottom tertile) and higher percentage of energy from dietary fat >35% of the total energy intake (OR: 1.73; 95% Cl: 1.06–2.83; *p* < 0.05; ref. 20–35%; Figure 1). Healthcare shift work compared to the daily work was associated with approximately 50% lower odds of the adherence to the ‘Lacto-ovo-vegetarian’ DP in the middle tertile (OR: 0.48; 95% Cl: 0.26–0.89; *p* < 0.05; ref. bottom tertile) and the higher level of the Polish-aMED^®^ score (OR: 0.57; 95% Cl: 0.33–0.98; *p* < 0.05; ref. lower level), as well as lower odds of the constants of mealtime (OR: 0.54; 95% Cl: 0.33–0.89; *p* < 0.05). No significant association between ‘Sweet-salty-snack-dairy’ DP and shift work was revealed (Figure 1).

## 4. Discussion

According to the authors’ best knowledge, this is the first study describing the association of dietary patterns, fat intake, and selected dietary habits including constants of mealtime with the mode of work among healthcare workers. The obtained results highlight that the Polish healthcare workers working in shifts did not have regular mealtime consumption, and made unhealthy food choices regarding the frequent consumption of processed meats, fats, and alcohol, which contributed to a high dietary fat intake of >35% of the total energy intake. In regards to the pro-healthy patterns, there was an inverse association of the shifts mode of work with the Polish-aMED^®^ score and the ‘Lacto-ovo-vegetarian’ pattern based on the plant food, fish, and non-sweetened dairy.

### 4.1. Mealtime among Healthcare Workers

Healthcare professionals are often obligated as a professional group to provide a model of healthy eating habits. In this study, it was observed that shift workers had approximately 50% less chance of consuming meals regularly compared to workers who worked in the daytime system. The study by Filipczuk et al. [29] showed that all surveyed nurses clearly confirmed that their behavior and eating attitudes may influence the shaping of nutritional and health attitudes of their charges [29]. In another study conducted by Sokołowska et al., it was observed that nurses declared the knowledge about the roles of healthy eating, but did not follow them by eating irregularly [30]. Additionally, half of the surveyed nurses emphasized that the shift work system hinders the regular nutrition of this professional group. It occurs more and more often that shift workers use catering and the box diet. A diverse, absorbing schedule often discourages systematic shopping and daily preparation of wholesome meals for the next days [30].

According to dietary recommendations, adults should consume 4–5 meals per day (polish recommendation [31,32,33]). The role of regular consumption of a smaller amount of food protects against hypoglycemia and consequently, hyperinsulinemia, which occurs after eating a heavy digestion meal eaten once during work [34]. In the conducted study, the consumption of at least 4 meals a day was declared by over 60% of healthcare workers, and the number of meals was not statistically significantly differentiated depending on the work mode of the respondents. Similarly, in the study by Sińska et al. [35] it was shown that, regardless of the nature of their work, 50% of the surveyed nurses eat 4 to 5 meals per day. In the current study, 8% of respondents declared eating 1–2 meals per day. In Bielak’s study, it was observed that 30% of nurses eat only 1–2 meals a day, whereas 25% of the nurses left home without eating their breakfast [36]. This model of nutrition may lead to a decrease in blood glucose levels, dizziness, increased fatigue, impaired concentration, decreased strength, endurance, and muscle regeneration. The consumption of two meals that are large in volume lengthens the digestive process. Long breaks between meals also lead to excessive secretion of ghrelin, which can consequently lead to overconsumption.

### 4.2. Unhealthy Food Choices and Fat Intake among Shift Healthcare Workers

In the current study it was noted that shift health workers consumed more processed meat and higher amounts of fat, especially of animal origin, compared to day care workers. This model of nutrition, combined with alcoholic beverages and fish, often in the form of canned fish, referred to as ‘Meat-fats-alcohol-fish’, was associated with a higher proportion of fat in the diet (over 35%). Consumption of saturated fatty acids (SFAs) is related to the development of cardiovascular disease [37]. SFAs increase the concentration of LDL, reduce the concentration of HDL, and increase the ratio of total cholesterol to HDL [38,39]. In 2015, the World Health Organization (WHO) classified the consumption of processed red meat—referring to compounds present in meat preserved by smoking, curing, salting, or the addition of chemical preservatives, including those contained in processed foods—as potentially carcinogenic [40,41]. Increased consumption of red meat is associated with premature death from various causes, increasing the risk of cancer, cardiovascular disease, and stroke [42,43,44,45]. There is also an increased risk of type 2 diabetes [45]. A high proportion of fat in the diet promotes weight gain. Both overweight and obesity in adulthood are associated with an increased risk of many types of cancers, coronary artery disease, and stroke, and were among the major risk factors influencing disability-adjusted life years in 2015 [46].

### 4.3. Pro-Healthy Dietary Patterns among Shift Healthcare Workers

The Mediterranean diet has been recognized as one of the healthiest models of nutrition. The Mediterranean diet is rich in fresh vegetables, especially greens and fruits, whole grains, pods, olive oil, lean fish and seafood, lean dairy products, herbs and spices, and small amounts of red wine [47]. In the current study, healthcare workers who worked shifts, compared to day workers, had about 50% less chance of achieving a higher level of the Polish-aMED^®^ score of the research. The Mediterranean diet is especially recommended due to its health benefits. The international project Healthy Aging: a Longitudinal study in Europe (HALE), confirmed the relationship between higher adherence to the Mediterranean diet and a reduced number of cardiovascular events [48]. Other studies confirmed that following the Mediterranean diet was associated with a reduced incidence of overweight and obesity, including abdominal obesity. According to a recent systematic review and meta-analysis, greater adherence to the Mediterranean diet appears to be inversely related to multiple cancer risk and overall cancer mortality [39]. The Mediterranean diet provides not only essentials vitamins and minerals but also large amounts of dietary fiber and polyphenols, which are included in fruits and vegetables, red wine, and olive oil. Phenolic compounds may act as modulators of gene expression and intracellular signaling cascades involved in cell function and protection [40,41].

In the current study, shift workers compared to day workers had about 50% less chance of adherence to the ‘Lacto-ovo-vegetarian’ pattern in the middle tertile. As in the case of the Mediterranean diet, unfortunately there is no similar research in this area, which makes comparative analyzes impossible. This plant-based pattern is associated with an increased consumption of dietary fiber, which has a number of health-promoting effects. Observational data show a reduction in morbidity and mortality from cardiovascular disease, type 2 diabetes and colorectal cancer in people who follow a diet rich in dietary fiber. In addition, the ‘Lacto-ovo-vegetarian’ pattern was also positively loaded by fish and non-sweetened dairy, which due to the content of beneficial polyunsaturated fatty acids and biopeptides are involved in the regulation of body weight, blood pressure and blood lipid profile [49].

### 4.4. Strengths and Limitations

This study included 445 participants who represented the healthcare environment. Additionally, it is the first study conducted among healthcare workers that divided them depending on the work system (day/shift). Thanks to this division, we were able to assess the differences in the consumption of selected groups of products and on their basis determine the nutrition patterns and calculate the Polish-aMED index, using two approaches—a priori and a posteriori. It is the first study in which holistic eating patterns, fat consumption, and selected eating habits were assessed in a group of healthcare professionals based on their work system. Three validated questionnaires were used in the study: (i) the 62-item Food Frequency Questionnaire FFQ-6^®^, (ii) the Quick Food Scan of the National Cancer Institute and the Percentage Energy from Fat Screener, and (iii) Godin Leisure Time Exercise Questionnaire, which allowed adequately assessing the diet, dietary fat intake, and level of physical activity of the participants. Notably, the questionnaires, which were used in this study, are widely used in studies assessing nutrition and lifestyle in various age groups, both for healthy people as well as patients with non-alcoholic fatty liver or cancer patients.

A limitation in the studies was the lack of a quantitative assessment of the diet using more precise methods, such as a 24-h interview or a diary of the current consumption. Thus, we cannot precisely assess the amount of calories consumed as well as the macro- and micronutrients depending on the type of work. The FFQ questionnaire provides the consumption over the last year, which is not precise. However, it should be emphasized that this is a questionnaire that allows for a retrospective assessment of food consumption over a longer period of time. Moreover, it is a relatively easy method, less labor-intensive for the respondent (it can be used online), and cheap. To increase the precision of the obtained results, the AICR quick fat intake questionnaire (variant FFQ) was used, which confirmed the results obtained with the FFQ-6 questionnaire (high coverage of the ‘Meat-fats-alcohol-fish’ pattern among shift system workers with a high fat consumption >35 %). Another limiting factor for the study was the fact that the data was not collected personally, but online. It was caused by the SARS-CoV2 virus pandemic and strongly limited/blocked the access to the wards. However, all participants were carefully instructed on how to complete the questionnaires correctly. Another limitation of the study was the representativeness of the group—it was a study involving healthcare workers only from Poland, but this group was sufficient to achieve the assumed the aim of study.

## 5. Conclusions

The obtained findings suggest the negative influence of shift work on regular mealtime consumption, and pro-healthy dietary patterns, including the Mediterranean diet, based on plant food, fish, and non-sweetened dairy among healthcare workers. Healthcare workers working in shifts more frequently consumed processed meats, animal fats, and alcohol, which contributed to an increased energy intake from dietary fat above 35% of the total energy intake. These unhealthy food choices could result from cheaper and more processed food purchases due to the thrifty living of shift healthcare workers.

Unhealthy dietary habits observed in shift healthcare workers can lead to negative health consequences and reduce the effectiveness of the work, which is associated with high responsibility. These study points indicate the need for nutritional education for healthcare workers, especially those working shifts, of the importance of regular meal consumption during a shift, and that imbalanced meals may have a harmful influence to their functioning at work during night shifts. There is a need to recommend shift healthcare workers to prepare and take pro-healthy meals and healthy snacks to work based on vegetables, fruits, grains and nuts, legumes, vegetable oils, and un-sweetened dairy, and the elimination of the consumption of processed food, including meats, animal fats, and alcohol. As a future recommendation, there should also be efforts to remove some of the institutional limitations of the healthcare system regarding increasing the access to healthy food choices, e.g., by providing healthy “vending machines” or restaurants with cooked meals for healthcare workers, as well as, ensuring more time and space for meal breaks during work time, also in night shifts.

## Figures and Tables

**Table 1:** Description of the socioeconomic status factors.

Socioeconomic Factors	Categories	Scoring
Place of residence	Village	1
	Town <50,000 inhabitants	2
	City 50,000–200,000 inhabitants	3
	City >200,000 inhabitants	4
Educational level	Primary	1
	Secondary	2
	Higher	3
Situation of household (self-declared)	We live very poorly—we do not have enough resources even for basic needs (food/clothing/housing fees)	1
	We live poorly—we only have enough resources for the cheapest food and clothing, but not for housing fees	2
	We live poorly—after paying the housing fees, we only have enough resources for the cheapest food and clothing	3
	We live very thriftily—we only have enough resources for basic needs (food/clothing/housing fees)	4
	We live thriftily—we have enough resources for everything	5
	We live well—we have enough resources for everything, without any special restrictions	6

Scoring—values assigned to the response categories.

**Table 2:** Sample characteristics (% or mean ± SD).

Variables	Total Sample	Mode of the Work		*p*-Value
		Shift	Daily	
Sample Size (n)	445	193	252	
Gender				
Men	20.0	16.6	22.6	0.1145
Women	80.0	83.4	77.4	
Age (years ^#^)	35.4 ± 10.9	34.0 ± 11.0	36.4 ±10.7	0.0188
<30.0	42.2	50.3	36.1	
30.0–39.9	27.4	22.8	31.0	0.0164
40.0–49.9	17.5	14.0	20.2	
≥50.0	12.8	13.0	12.7	
Place of residence				
Village	14.8	14.5	15.1	
Town <50,000 inhabitants	14.6	17.6	12.3	0.0872
City (50,000–200,000 inhabitants)	18.4	21.8	15.9	
City (>200,000 inhabitants)	52.1	46.1	56.7	
Educational level				
Primary	0.5	0.5	0.4	
Secondary	6.8	7.8	6.0	0.7256
Higher	92.8	91.7	93.7	
Situation of household				
We live poorly—after paying the housing fees, we have enough resources only for the cheapest food and clothing	1.6	1.6	1.6	
We live very thriftily—we have enough resources only for basic needs (food/clothing/housing fees)	9.7	13.0	7.2	0.0495
We live thriftily—so we have enough resources for everything	33.5	37.0	30.8	
We live well—we have enough resources for everything, without any special restrictions	55.2	48.4	60.4	
Socioeconomic status (SES Index, points ^#^)	11.4 ± 1.5	11.2 ± 1.4	11.6 ± 1.5	0.0045
Low (3–6)	0.5	0.0	0.8	
Average (7–10)	26.5	29.8	24.0	0.1922
High (11–13)	73.0	70.2	75.2	
BMI (kg/m^2 #^)	24.2 ± 3.0	24.2 ± 3.2	24.3 ± 2.9	0.7891
Underweight (<18.5)	1.4	2.6	0.4	
Normal weight (18.5–24.9)	55.0	54.4	55.4	
Overweight (25.0–29.9)	40.3	38.9	41.4	0.1974
Obesity (≥30)	3.4	4.1	2.8	
Chronic diseases	38.2	33.5	41.8	0.0772
Taking medication >1 year	31.5	25.9	35.7	0.0272
Physical activity ^1^				
GLTEQ (summary points ^#^)	36.3 ± 28.4	36.4 ± 29.5	36.3 ± 27.2	0.8274
GLTEQ (strenuous and moderate points ^#^)	27.8 ± 24.4	28.1 ± 25.1	27.4 ± 23.6	0.9711
Insufficiently active (<14 points)	30.3	30.7	29.9	
Moderately active (14–23 points)	20.3	20.5	20.1	0.9780
Active (≥24 points)	49.3	48.8	50.0	
Alcohol drinking				
Within the 12 last months	35.4	33.9	36.5	0.5623
Within the last 10 years	75.2	74.9	75.4	0.8987
Smoking status (smoker ^2^)	54.7	55.4	54.2	0.7919
Current smoker				
No	83.5	83.8	83.3	
Yes, up to 5 cigarettes/day	6.3	6.3	6.3	
Yes, up to 10 cigarettes/day	6.5	7.3	6.0	0.7356
Yes, up to 20 cigarettes/day	2.7	1.6	3.6	
Yes, up >20 cigarettes/day	0.9	1.0	0.8	
Vitamin/mineral supplements use ^3^	70.8	65.3	75.0	0.0255

SES—socioeconomic status calculated on the basis of place of residence, education level, and self-declared situation of household (description in the Materials and Methods section); BMI—body mass index; ^1^ data for n = 296; ^2^ ever-smoker (current and/or former smoker); ^3^ self-declared use of vitamin and/or mineral supplements within the last 12 months; %—sample percentage; ^#^ mean and standard deviation (SD); *p*-value—level of significance verified with chi^2^ test (categorical variables) or Kruskal–Wallis’ test (continuous variables); *p* < 0.05—statistically significant.

**Table 3:** Factor loadings for food groups in Principal Component Analysis (PCA)-derived dietary patterns and the Pearson’s correlation coefficients for food groups in the Polish-a MED^®^ among healthcare workers (n = 445).

Food Groups	PCA-Derived Dietary Patterns			Polish-aMED^®^ Score
	‘Sweet-Salty-Snack-Dairy’	‘Lacto-Ovo-Vegetarian’	‘Meat- Fats-Alcohol-Fish’	
Sweetened milk drinks and flavored cheese	**0.72**	0.06	0.07	−0.03
Salty snacks	**0.66**	−0.07	0.19	−0.06
Sugar, honey, and sweets	**0.62**	−0.08	0.23	−0.15 *
Refined grains	**0.52**	0.03	0.21	−0.08
Breakfast cereals	**0.52**	0.01	−0.14	0.00
Milk, fermented milk drinks, and cheese curd	**0.49**	**0.43**	−0.19	0.27 *
Cheese	**0.45**	0.22	0.19	0.11 *
Sweetened beverages	**0.40**	−0.28	0.28	−0.22 *
Animal fats	**0.34**	0.14	**0.55**	−0.16 *
Potatoes	**0.31**	0.23	0.29	0.08
Vegetables	0.00	**0.77**	−0.04	**0.69** *
Fruits	0.07	**0.65**	−0.06	**0.51** *
Whole grains	−0.02	**0.65**	−0.05	**0.58** *
Legumes	−0.11	**0.62**	0.12	**0.49** *
Nuts and seeds	−0.05	**0.62**	0.16	**0.45** *
Vegetable oils (including olive oil)	0.15	**0.44**	**0.31**	**0.30** *
Eggs	0.23	**0.41**	0.20	0.20 *
Processed meats	0.22	−0.14	**0.69**	**−0.31** *
Other fats (margarine, mayonnaise, dressings)	0.26	−0.04	**0.61**	−0.13 *
White meat	0.09	0.13	**0.60**	0.02
Alcoholic drinks	0.03	0.03	**0.60**	−0.03
Fish	−0.12	0.28	**0.54**	**0.28** *
Fruit, vegetable or vegetable-fruit juices	0.24	−0.02	0.20	−0.09
Ratio of vegetable oils to animal fats	NA	NA	NA	**0.18** *
Share in explaining the variance (%)	18.6	12.5	7.3	NA

Polish-aMED^®^—Polish-adapted Mediterranean diet (range of points: 0–8); NA—not applied; bolded values are marked for the main components of PCA-derived dietary patterns with absolute loadings ≥ 0.3 and for components of the Polish-aMED^®^ score; * *p* < 0.05, test of significance for Pearson’s correlation coefficients.

**Table 4:** Food choices and dietary fat intake in association with the mode of the work among healthcare workers (% or mean ± SD).

Variables	Total Sample	Mode of the Work		*p*-Value
		Shift	Daily	
Sample Size	445	193	252	
PCA-derived dietary patterns (tertiles)				
‘Sweet-salty-snack-dairy’				
Bottom	33.3	28.5	36.9	
Middle	33.3	35.8	31.3	0.1752
Upper	33.5	35.8	31.7	
‘Lacto-ovo-vegetarian’				
Bottom	33.3	40.9	27.4	
Middle	33.5	26.4	38.9	0.0038
Upper	33.3	32.6	33.7	
‘Meat- fats-alcohol-fish’				
Bottom	33.5	27.5	38.1	
Middle	33.0	30.6	34.9	0.0030
Upper	33.5	42.0	27.0	
Polish-amed^®^ score (points) ^#^	3.9 ± 1.7	3.5 ± 1.8	4.1 ± 1.6	0.0001
Levels (points)				
Lower (0–4)	66.7	73.6	61.5	
Higher (5–8)	33.3	26.4	38.5	0.0074
Total fat intake (g) ^#^	3.4 ± 4.8	4.1 ± 5.4	2.9 ± 4.2	0.0395
Regular fat intake (g) ^#^	3.1 ± 4.5	3.7 ± 5.0	2.7 ± 4.0	0.1546
Percentage energy from dietary fat ^#^	35.4 ± 5.9	36.4 ± 6.4	34.6 ± 5.4	0.0013
20–35%	57.8	49.7	63.9	
>35%	42.2	50.3	36.1	0.0028
Number of meals				
1–2	8.3	9.3	7.5	
3	27.9	29.0	27.0	0.7367
4	44.3	41.5	46.4	
≥5	19.6	20.2	19.0	
Constants of mealtime (yes)	54.6	44.0	62.7	<0.0001
Special diet or intake restrictions	40.8	34.2	45.8	0.0135
Overall decrease in food consumption	74.6	72.5	76.2	0.3805
Restriction in consumption of:				
Dairy	29.1	21.8	34.7	0.0030
Fish	16.9	14.5	18.8	0.2323
Fruits	9.7	5.7	12.7	0.0128
Raw vegetables	5.2	4.2	6.0	0.4003
Fats	54.2	50.3	57.2	0.1460
Foods in high fat content	64.3	60.1	67.5	0.1085
Sugar and sweets	70.7	66.1	74.1	0.0683
Potatoes and cereals	29.9	21.8	36.1	0.0011
Meat and meat products	42.9	33.2	50.4	0.0003

Polish-aMED^®^—Polish-adapted Mediterranean Diet (range of points: 0–8); %—sample percentage; ^#^ mean and standard deviation (SD); *p*-value—level of significance assessed by chi^2^ test (categorical variables) or Kruskal–Wallis’ test (continuous variables); *p* < 0.05.

## Data Availability

Not applicable.

## Supplementary Materials

The following supporting information can be downloaded at: https://www.mdpi.com/article/10.3390/nu14204327/s1, Table S1: Description of 23 food groups aggregated: data based on the FFQ-6 questionnaire; Table S2: Description of food groups for the Polish-adapted Mediterranean Diet^®^ score (0–8 points) calculation—data for the initial control sample (n = 412); Table S3: The mean (95%CI) of the frequency of food consumption by dietary patterns among Polish healthcare workers (times/day); Table S4: The sample characteristics by dietary patterns among Polish healthcare workers (%) or mean (SD); Table S5: The mean (SD) of the food consumption by mode of the work among Polish healthcare workers; Table S6: Fat intake, food consumption, and dietary patterns in association with the percentage of energy from dietary fat among Polish healthcare workers (% or mean (SD).

## References

[1] Gacek M. (2018). Selected Aspects of Life Style of Women with Secondaryand Higher Education Employed as Shift Workers. Med. Ogólna Nauk. O Zdrowiu.

[2] Kuleta A. (2016). Wpływ pracy zmianowej na wystąpienie zmian patofizjologicznych—Przegląd literatury. Forum Zaburzeń Metab..

[3] Costa G. (2003). Shift Work and Occupational Medicine: An Overview. Occup. Med..

[4] Leka S., Jain A., Orlak K. (2013). Zdrowa Praca Zagrozenia Psychospoleczne w Srodowisku Pracy i ich Wplyw na Zdrowie.

[5] Lewandowski J. (2010). Epidemiologia Nadciśnienia Tętniczego Oraz Badanie Chorego Na Nadciśnienie Tętnicze. Przew Lek.

[6] Dzierżewicz Z., Balwierz R., Marciniak D., Sarecka-Hujar B., Delijewski M., Dolińska B. (2018). Plejotropowe Działanie Melatoniny. Med. Rodz..

[7] Pawlak J., Pawlak B., Zalewski P., Bitner A. (2013). Praca Zmianowa a Powstawanie Chorób Układu Sercowonaczyniowego w Kontekście Regulacji Normatywnej. Hygeia Public Health.

[8] Liu W., Zhou Z., Dong D., Sun L., Zhang G. (2018). Sex Differences in the Association between Night Shift Work and the Risk of Cancers: A Meta-Analysis of 57 Articles. Dis. Markers.

[9] Roenneberg T., Merrow M. (2003). The Network of Time: Understanding the Molecular Circadian System. Curr. Biol..

[10] Waterhouse J., Minors D., Atkinson G., Benton D. (1997). Chronobiology and Meal Times: Internal and External Factors. Br. J. Nutr..

[11] Keogh K. (2014). Shift Work and Vending Machines to Blame for Poor Workplace Diet: Nurses Are Struggling with Their Weight as the Pressures of the Job Leave Little Time for Healthy Eating, a Nursing Standard Survey Reveals. Kat Keogh Reports. Nurs. Stand..

[12] Hulsegge G., Boer J.M., van der Beek A.J., Verschuren W.M., Sluijs I., Vermeulen R., Proper K.I. (2016). Shift Workers Have a Similar Diet Quality but Higher Energy Intake than Day Workers. Scand. J. Work. Environ. Health.

[13] Fernandes J.d.C., Portela L.F., Rotenberg L., Griep R.H. (2013). Working Hours and Health Behaviour among Nurses at Public Hospitals. Rev. Lat. Am. Enferm..

[14] Newby P.K., Tucker K.L. (2004). Empirically Derived Eating Patterns Using Factor or Cluster Analysis: A Review. Nutr. Rev..

[15] Canuto R., Garcez A., Spritzer P.M., Olinto M.T.A. (2021). Associations of Perceived Stress and Salivary Cortisol with the Snack and Fast-Food Dietary Pattern in Women Shift Workers. Stress.

[16] Khorasaniha R., Siassi F., Khajehnasiri F., Qorbani M., Sotoudeh G. (2020). Dietary Patterns in Relation to Inflammation in Shift Workers. BMJ Mil Health.

[17] Mota M.C., Waterhouse J., De-Souza D.A., Rossato L.T., Silva C.M., Araújo M.B.J., Tufik S., de Mello M.T., Crispim C.A. (2014). Sleep Pattern Is Associated with Adipokine Levels and Nutritional Markers in Resident Physicians. Chronobiol. Int..

[18] Mortaş H., Bilici S., Öztürk H., Karakan T. (2022). Changes in Intestinal Parameters and Their Association with Dietary Patterns in Rotational Shift Workers. Chronobiol. Int..

[19] Niedzwiedzka E., Wadolowska L., Kowalkowska J. (2019). Reproducibility of A Non-Quantitative Food Frequency Questionnaire (62-Item FFQ-6) and PCA-Driven Dietary Pattern Identification in 13–21-Year-Old Females. Nutrients.

[20] Krusinska B., Hawrysz I., Wadolowska L., Slowinska M., Biernacki M., Czerwinska A., Golota J. (2018). Associations of Mediterranean Diet and a Posteriori Derived Dietary Patterns with Breast and Lung Cancer Risk: A Case-Control Study. Nutrients.

[21] Stachowska E., Ryterska K., Maciejewska D., Banaszczak M., Milkiewicz P., Milkiewicz M., Gutowska I., Ossowski P., Kaczorowska M., Jamioł-Milc D. (2016). Nutritional Strategies for the Individualized Treatment of Non-Alcoholic Fatty Liver Disease (NAFLD) Based on the Nutrient-Induced Insulin Output Ratio (NIOR). Int. J. Mol. Sci..

[22] Armitage C.J., Conner M. (2001). Efficacy of the Theory of Planned Behaviour: A Meta-Analytic Review. Br. J. Soc. Psychol..

[23] National Cancer Institute, Division of Cancer Control and Population Sciences—Percentage En-Ergy from Fat Screener: Scoring Procedures. https://Epi.Grants.Cancer.Gov/Diet/Screeners/Fat/Scoring.Html.

[24] Katherine S.T., Linda E. (2001). Cleveland Results from USDA’s 1994-96 Diet and Health Knowledge Survey.

[25] Stasiewicz B., Wadolowska L., Biernacki M., Slowinska M.A., Stachowska E. (2022). Dietary Fat Intake: Associations with Dietary Patterns and Postmenopausal Breast Cancer-A Case-Control Study. Cancers.

[26] (2011). Gaston Godin The Godin Shephard Leisure Time Physical Activity Questionnaire. Health Fit. J. Can..

[27] Krupa-Kozak U., Drabińska N., Jarocka-Cyrta E. (2017). The Effect of Oligofructose-Enriched Inulin Supplementation on Gut Microbiota, Nutritional Status and Gastrointestinal Symptoms in Paediatric Coeliac Disease Patients on a Gluten-Free Diet: Study Protocol for a Pilot Randomized Controlled Trial. Nutr. J..

[28] Ahmed S., Rahman T., Ripon M.S.H., Rashid H.-U., Kashem T., Md Ali M.S., Khor B.-H., Khosla P., Karupaiah T., Daud Z.A.M. (2021). A Food Frequency Questionnaire for Hemodialysis Patients in Bangladesh (BDHD-FFQ): Development and Validation. Nutrients.

[29] Filipczuk A., Wrońska I. (2003). Promocja Zdrowia Wśród Pracowników Ochrony Zdrowia. Ann. UMCS.

[30] Sokołowska B., Samoszuk T., Piaszczyk D. (2003). Styl Życia a Odżywianie Pielęgniarek Jako Jeden z Wyznaczników Programu Promocji Zdrowia. Ann. UMCS.

[31] Gawęcki J., Mossor-Pietraszewska T. (2014). Kompendium Wiedzy o Żywności, Żywieniu i Zdrowiu.

[32] Jarosz M., Respondek W., Wolnicka W., Jarosz M. (2012). Zalecenia Dotyczące Żywienia i Aktywności Fizycznej.

[33] Jeżewska-Zychowicz M. (2007). Zachowania Żywieniowe i Ich Uwarunkowania.

[34] Łokieć K., Górska-Ciebiada M. (2020). Nutritional Behaviours of Shift Workers. Med. Ogólna Nauk. O Zdrowiu.

[35] Sińska B., Kucharska A., Dykowska G. (2018). Zakład Ekonomiki i Prawa, Warszawski Uniwersytet Medyczny, Warszawa, Polska Wpływ Systemu Zmianowego Pracy Pielęgniarek Na Ich Sposób Odżywiania i Aktywność Fizyczną. Zdr. Publiczne Zarządzanie.

[36] Siedlecka J. (2006). Selected work-related health problems in drivers of public transport vehicles. Med. Pr..

[37] Kochan Z., Karbowska J., Babicz-Zielińska E. (2010). Dietary trans-fatty acids and metabolic syndrome. Postep. Hig. Med. Dosw. Online.

[38] Beunza J.-J., Toledo E., Hu F.B., Bes-Rastrollo M., Serrano-Martínez M., Sánchez-Villegas A., Martínez J.A., Martínez-González M.A. (2010). Adherence to the Mediterranean Diet, Long-Term Weight Change, and Incident Overweight or Obesity: The Seguimiento Universidad de Navarra (SUN) Cohort. Am. J. Clin. Nutr..

[39] Schwingshackl L., Schwedhelm C., Galbete C., Hoffmann G. (2017). Adherence to Mediterranean Diet and Risk of Cancer: An Updated Systematic Review and Meta-Analysis. Nutrients.

[40] Barrajón-Catalán E., Herranz-López M., Joven J., Segura-Carretero A., Alonso-Villaverde C., Menéndez J.A., Micol V., Camps J. (2014). Molecular Promiscuity of Plant Polyphenols in the Management of Age-Related Diseases: Far Beyond Their Antioxidant Properties. Oxidative Stress and Inflammation in Non-Communicable Diseases—Molecular Mechanisms and Perspectives in Therapeutics.

[41] Joven J., Micol V., Segura-Carretero A., Alonso-Villaverde C., Menéndez J.A., for the Bioactive Food Components Platform (2014). Polyphenols and the Modulation of Gene Expression Pathways: Can We Eat Our Way Out of the Danger of Chronic Disease?. Crit. Rev. Food Sci. Nutr..

[42] Bouvard V., Loomis D., Guyton K.Z., Grosse Y., Ghissassi F.E., Benbrahim-Tallaa L., Guha N., Mattock H., Straif K. (2015). Carcinogenicity of Consumption of Red and Processed Meat. Lancet Oncol..

[43] Zeraatkar D., Han M.A., Guyatt G.H., Vernooij R.W.M., El Dib R., Cheung K., Milio K., Zworth M., Bartoszko J.J., Valli C. (2019). Red and Processed Meat Consumption and Risk for All-Cause Mortality and Cardiometabolic Outcomes: A Systematic Review and Meta-Analysis of Cohort Studies. Ann. Intern. Med..

[44] Han M.A., Zeraatkar D., Guyatt G.H., Vernooij R.W.M., El Dib R., Zhang Y., Algarni A., Leung G., Storman D., Valli C. (2019). Reduction of Red and Processed Meat Intake and Cancer Mortality and Incidence: A Systematic Review and Meta-Analysis of Cohort Studies. Ann. Intern. Med..

[45] Vernooij R.W.M., Zeraatkar D., Han M.A., El Dib R., Zworth M., Milio K., Sit D., Lee Y., Gomaa H., Valli C. (2019). Patterns of Red and Processed Meat Consumption and Risk for Cardiometabolic and Cancer Outcomes: A Systematic Review and Meta-Analysis of Cohort Studies. Ann. Intern. Med..

[46] Gakidou E., Afshin A., Abajobir A.A., Abate K.H., Abbafati C., Abbas K.M., Abd-Allah F., Abdulle A.M., Abera S.F., Aboyans V. (2017). Global, Regional, and National Comparative Risk Assessment of 84 Behavioural, Environmental and Occupational, and Metabolic Risks or Clusters of Risks, 1990–2016: A Systematic Analysis for the Global Burden of Disease Study 2016. Lancet.

[47] Dominguez L.J., Di Bella G., Veronese N., Barbagallo M. (2021). Impact of Mediterranean Diet on Chronic Non-Communicable Diseases and Longevity. Nutrients.

[48] Martínez-González M.A., García-López M., Bes-Rastrollo M., Toledo E., Martínez-Lapiscina E.H., Delgado-Rodriguez M., Vazquez Z., Benito S., Beunza J.J. (2010). Mediterranean Diet and the Incidence of Cardiovascular Disease: A Spanish Cohort. Nutr. Metab. Cardiovasc. Dis..

[49] Ma W., Nguyen L.H., Song M., Wang D.D., Franzosa E.A., Cao Y., Joshi A., Drew D.A., Mehta R., Ivey K.L. (2021). Dietary Fiber Intake, the Gut Microbiome, and Chronic Systemic Inflammation in a Cohort of Adult Men. Genome Med..

