# Supplementary material for: Unhealthy Food Choices among Healthcare Shift Workers: A Cross-Sectional Study
Source: Nutrients. 2022 Oct 16;14(20):4327. doi: 10.3390/nu14204327 (PMC9611829; doi:10.3390/nu14204327)
Supplement: Supplementary file 1 [file nutrients-14-04327-s001.zip › nutrients-1977765-supplementary.pdf]

Table S1. Description of 23 food groups aggregated: data based on the FFQ-6 questionnaire [Niedzwiedzka et al. 2019].

No	Food groups	Food groups description (food items)
1	Sugar, honey and sweets	Sugar added to beverages, such as tea, coffee, etc.;<br>Honey added to dishes and added to beverages;<br>Chocolates, chocolate sweets and chocolate bars, sugar confectionery (boiled sweets, hard caramels, jellied sweets, fudge, etc.), baked confectionery (biscuits, cream cakes, fruit cakes, sponge cakes, cheesecakes, doughnuts, poppy-seed cakes, muffins, croissants, etc.), ice-creams and custard.
2	Milk and milk beverages – natural and cheese curds	Milk and natural milk beverages (yoghurt, kefir, buttermilk), porridge, etc.<br>Cheese curd, natural cottage cheese, soft cheese, mozzarella, cottage cheese with herbs, etc.
3	Milk beverages – sweetened and flavoured cheese curds	Fruit yoghurts, yoghurts with chocolate flakes, flavoured buttermilk, hot chocolate, etc.<br>Flavoured curds (with fruit, chocolate, vanilla), etc.
4	Cheese	Hard cheese, blue cheese, processed cheese, cheese spreads, etc.
5	Eggs and egg dishes	Scrambled eggs, omelette, egg salad, cooked eggs.
6	Whole grains	Wholemeal wheat or rye bread, seeded loafs, pumpernickel, wholemeal cracker bread, etc.<br>Buckwheat groats, barley, brown rice, wholemeal pasta, etc.
7	Refined grains	Semolina, milled barley, pasta, white rice, rice flakes, etc.
8	Breakfast cereals	Prepared breakfast cereals, unboiled milk additives, e.g. muesli, cornflakes with or without additives
9	Vegetable based oil	Vegetable oils;<br>Olive oil.
10	Animal fats	Butter;<br>Lard, pork fat, etc.<br>Cream (single, double, sour, used as an ingredient or added to beverages).
11	Other fats	all kinds of margarine in cubes (for frying, baking), margarine in cups (for spreading)<br>mayonnaise, salad dressings
12	Fruits	All kinds of fruits.
13	Vegetables	All kinds of vegetables (potatoes not included).
14	Legumes	Fresh and tinned legumes (corn, green peas, green beans, etc.);<br>Dry and processed pulses (beans (fava, butter kidney, broad, French, green), soya, peas, chickpea and processed pulses (baked beans, hummus, other bread spreads)).
15	Potatoes	Boiled, baked, French fries, potato rosti, gnocchi, etc.
16	Nuts and seeds	Peanuts, hazelnuts, walnuts, cashews, coconuts, chestnuts, peanut butter, chocolate-nut spread, etc.<br>Pumpkin seeds, sesame seeds, sunflower seeds, wheat germs, wheat bran, etc.
17	Processed meats	Sausages, bacon, reconstituted meat (sausages, meat loaf, hot-dogs, smoked sausages, bacon, etc.)<br>High quality cured meats (ham, poultry and pork-beef good quality cold meats, etc.)<br>Offal products (liver, blood sausage, sweetbread, liver pate, etc.).
18	White meat	Poultry and rabbit.
19	Fish	Lean fish (pollock, cod, perch, hake, carp to 1 kg, tuna, panga, trout, etc.)<br>Oily fish (salmon, sardines, herring, mackerel, eel, large carp, etc.).
20	Fruit, vegetable or vegetable-fruit juices	Fruit juices and nectars (mixed fruit juice, orange, grapefruit, apple, pear, grape, blackcurrant, cherry juice)<br>Vegetable and vegetable-fruit juices (mixed vegetable juice, tomato, carrot and carrot-fruit juice).
21	Sweetened beverages	All kinds of sweetened beverages.
22	Alcoholic drinks	All kinds of alcohol, including beer, wine, drinks, vodka, and spirits
23	Salty snacks	Chipsy, crackers, salted sticks, salted peanuts, etc.

Table S2. Description of food groups for the Polish-adapted Mediterranean Diet® score (0-8 points) calculation – data for the Initial control sample (n=412) [Krusinska et al. 2018].

**Food groups/food items**	**Frequency of consumption** (times/day)*		**Criteria for 1 point**
	**Mean (95% CI)**	**Median**	
**VEGETABLES**: all kinds of vegetables: cruciferous vegetables (cabbages, brussel sprouts, cauliflower, broccoli, kale, etc.), yellow-orange vegetables (carrots, peppers, etc.), leafy green vegetables (spinach, chicory, lettuce, rocket, leek, celery, parsley, etc.), tomatoes, gourds and squashes (fresh cucumber, marrow, courgettes, pumpkins, aubergines, etc.), root vegetables and other (parsnip, beetroots, onion, garlic, celeriac, radishes, turnip, salads and mixed vegetables, etc.)	1.064 (1.010; 1.117)	1.000	Greater than median intake (times/day)*
**FRUIT**: all kinds of fruit: stone fruit (apricots, cherries, nectarines, peaches, plums, grapes, etc.), kiwi and citrus fruit (kiwi, oranges, mandarins, grapefruit, lemons, pomelos, etc.), tropical fruits (pineapples, watermelon, melons, fresh dates and figs, etc.), berries (strawberries, raspberries, blackberries, blueberries, redcurrants, blackcurrants, etc.), bananas, apples, pears, avocado	0.917 (0.867; 0.967)	1.000	Greater than median intake (times/day)*
**WHOLE GRAINS**: wholemeal wheat or rye bread, seeded loafs, pumpernickel, wholemeal groats, wholemeal rice, wholemeal pasta	0.767 (0.703; 0.832)	0.671	Greater than median intake (times/day)*
**FISH**: lean fish (ollock, cod, perch, hake, carp to 1 kg, tuna, panga, trout, etc.) oily fish (salmon, sardines, herring, mackerel, eel, large carp, etc.)	0.268 (0.238; 0.297)	0.200	Greater than median intake (times/day)*
**LEGUMES**: fresh and tinned legumes (corn, green peas, green beans, etc.), dry and processed pulses beans (fava, broad, French, green), soya, peas, chickpea and processed pulses (baked beans, hummus, other bread spreads)	0.208 (0.181; 0.235)	0.125	Greater than median intake (times/day)*
**NUTS** and **SEEDS**: peanuts, hazelnuts, walnuts, almonds, pistachios, cashews, coconuts, chestnuts, etc., pumpkin seeds, sesame seeds, sunflower seeds, wheat germs, etc.	0.281 (0.239; 0.323)	0.100	Greater than median intake (times/day)*
**RATIO of VEGETABLES OILS** (rapeseed oil, sunflower oil, linseed oil, olives) **to ANIMAL FAT** (butter, cream, lard) instead of RATIO of MONOUNSATURATED to SATURATED FAT	1.745 (1.231; 2.258)	0.500	Greater than median intake (times/day)*
**PROCESED MEAT**: sausages, bacon, reconstituted meat (sausages, meat loaf, hot-dogs, smoked sausages, bacon, etc.), high quality cured meats (ham, poultry and pork-beef good quality cold meats, etc.), offal products (liver, blood sausage, sweetbread, liver pate, etc.).	1.151 (1.081; 1.221)	1.167	Lower than median intake (times/day)*

*food frequency consumption was expressed as a times/day after assigning the values for categories of frequencies as follows: 'never or almost never'=0; 'once a month or less'=0.025; 'several times a month'=0.1; 'several times a week'=0.571; 'daily'=1; 'several times a day'=2; 95% CI – 95% confidence interval.

Table S3. The mean (95%CI) of the frequency of food consumption by dietary patterns among Polish health care workers (times/day).

Food groups[#]	Dietary Patterns (tertiles/levels)										
	'Sweet-salty-snack-dairy'			'Lacto-ovo-vegetarian'			'Meat- fats-alcohol-fish'			Polish-aMED®	
	bottom	middle	upper	bottom	middle	upper	bottom	middle	upper	lower	higher
Size (n)	148	148	149	148	149	148	149	147	149	297	148
Sugar, honey and sweets	0.5 (0.4, 0.5)	1.1 (0.9, 1.2)	2.3 (2.0, 2.6)	1.4 (1.2, 1.7)	1.3 (1.1, 1.5)	1.0 (0.9, 1.2)	0.9 (0.8, 1.1)	1.2 (1.0, 1.4)	1.7 (1.4, 2.0)	1.4 (1.2, 1.6)	1.0 (0.9, 1.2)
Milk and milk beverages – natural and cheese curds	0.5 (0.4, 0.6)	1.0 (0.9, 1.2)	1.5 (1.3, 1.6)	0.6 (0.5, 0.7)	1.0 (0.9, 1.1)	1.4 (1.3, 1.6)	1.2 (1.1, 1.4)	0.9 (0.8, 1.0)	0.9 (0.8, 1.0)	0.9 (0.8, 1.0)	1.3 (1.1, 1.4)
Milk beverages – sweetened and flavoured cheese curds	0.0 (0.0, 0.1)	0.2 (0.1, 0.2)	0.6 (0.5, 0.7)	0.3 (0.2, 0.3)	0.3 (0.2, 0.3)	0.3 (0.2, 0.4)	0.3 (0.2, 0.4)	0.2 (0.2, 0.3)	0.3 (0.3, 0.4)	0.3 (0.2, 0.3)	0.3 (0.2, 0.3)
Cheese	0.2 (0.1, 0.2)	0.4 (0.3, 0.5)	0.7 (0.6, 0.8)	0.3 (0.2, 0.4)	0.4 (0.3, 0.5)	0.6 (0.5, 0.6)	0.4 (0.3, 0.5)	0.4 (0.3, 0.4)	0.5 (0.4, 0.6)	0.4 (0.4, 0.5)	0.4 (0.4, 0.5)
Eggs and egg dishes	0.4 (0.3, 0.5)	0.5 (0.4, 0.6)	0.6 (0.5, 0.7)	0.3 (0.3, 0.4)	0.5 (0.4, 0.5)	0.7 (0.6, 0.8)	0.4 (0.4, 0.5)	0.5 (0.4, 0.5)	0.6 (0.5, 0.6)	0.5 (0.4, 0.5)	0.6 (0.5, 0.7)
Whole grains	0.8 (0.7, 0.9)	1.0 (0.9, 1.1)	0.9 (0.7, 1.0)	0.4 (0.3, 0.4)	0.9 (0.8, 1.0)	1.4 (1.3, 1.5)	0.9 (0.8, 1.1)	0.9 (0.8, 1.0)	0.9 (0.7, 1.0)	0.6 (0.6, 0.7)	1.4 (1.3, 1.5)
Refined grains	0.2 (0.2, 0.3)	0.6 (0.5, 0.6)	1.1 (0.9, 1.2)	0.6 (0.5, 0.7)	0.7 (0.6, 0.7)	0.6 (0.5, 0.7)	0.5 (0.4, 0.6)	0.6 (0.5, 0.6)	0.8 (0.7, 0.9)	0.6 (0.5, 0.7)	0.6 (0.5, 0.7)
Breakfast cereals	0.0 (0.0, 0.0)	0.1 (0.1, 0.1)	0.4 (0.3, 0.4)	0.1 (0.1, 0.2)	0.2 (0.1, 0.2)	0.1 (0.1, 0.2)	0.2 (0.1, 0.3)	0.1 (0.1, 0.2)	0.2 (0.1, 0.2)	0.1 (0.1, 0.2)	0.2 (0.1, 0.2)
Vegetable based oil	0.4 (0.3, 0.4)	0.4 (0.4, 0.5)	0.5 (0.5, 0.6)	0.3 (0.2, 0.3)	0.4 (0.3, 0.5)	0.6 (0.6, 0.7)	0.3 (0.3, 0.4)	0.4 (0.3, 0.5)	0.6 (0.5, 0.7)	0.4 (0.3, 0.4)	0.6 (0.5, 0.7)
Animal fats	0.5 (0.4, 0.6)	0.6 (0.5, 0.7)	1.0 (0.9, 1.1)	0.7 (0.6, 0.8)	0.7 (0.6, 0.8)	0.8 (0.7, 1.0)	0.4 (0.3, 0.4)	0.7 (0.6, 0.8)	1.1 (1.0, 1.3)	0.8 (0.7, 0.9)	0.6 (0.5, 0.7)
Other fats	0.2 (0.1, 0.3)	0.2 (0.1, 0.3)	0.4 (0.4, 0.5)	0.4 (0.3, 0.4)	0.2 (0.2, 0.3)	0.3 (0.2, 0.3)	0.1 (0.1, 0.1)	0.2 (0.2, 0.3)	0.5 (0.4, 0.6)	0.3 (0.3, 0.4)	0.2 (0.1, 0.2)
Fruits	0.6 (0.6, 0.7)	0.8 (0.8, 0.9)	0.8 (0.7, 0.9)	0.4 (0.3, 0.4)	0.8 (0.7, 0.8)	1.1 (1.0, 1.2)	0.8 (0.7, 0.9)	0.8 (0.7, 0.9)	0.7 (0.6, 0.8)	0.6 (0.6, 0.7)	1.0 (0.9, 1.1)
Vegetables	0.9 (0.8, 1.0)	1.0 (0.9, 1.1)	0.9 (0.8, 1.1)	0.4 (0.3, 0.5)	0.9 (0.8, 1.0)	1.5 (1.4, 1.6)	0.9 (0.8, 1.0)	0.9 (0.8, 1.0)	0.9 (0.8, 1.0)	0.7 (0.6, 0.7)	1.4 (1.3, 1.5)
Legumes	0.6 (0.5, 0.8)	0.5 (0.4, 0.6)	0.4 (0.4, 0.5)	0.2 (0.1, 0.2)	0.4 (0.3, 0.5)	1.0 (0.9, 1.1)	0.5 (0.4, 0.6)	0.6 (0.4, 0.7)	0.6 (0.4, 0.7)	0.3 (0.3, 0.4)	0.9 (0.8, 1.0)
Potatoes	0.2 (0.2, 0.3)	0.4 (0.3, 0.4)	0.5 (0.4, 0.6)	0.3 (0.2, 0.4)	0.4 (0.3, 0.5)	0.4 (0.4, 0.5)	0.3 (0.2, 0.3)	0.3 (0.3, 0.4)	0.5 (0.5, 0.6)	0.4 (0.3, 0.4)	0.4 (0.3, 0.5)
Nuts and seeds	0.9 (0.7, 1.0)	0.6 (0.5, 0.7)	0.7 (0.6, 0.8)	0.3 (0.2, 0.4)	0.6 (0.5, 0.6)	1.3 (1.1, 1.5)	0.6 (0.5, 0.7)	0.7 (0.5, 0.8)	0.9 (0.7, 1.0)	0.5 (0.5, 0.6)	1.1 (0.9, 1.3)
Processed meats	0.5 (0.3, 0.6)	0.5 (0.4, 0.6)	0.8 (0.7, 1.0)	0.7 (0.6, 0.9)	0.6 (0.5, 0.7)	0.4 (0.3, 0.5)	0.2 (0.1, 0.2)	0.4 (0.4, 0.5)	1.2 (1.1, 1.3)	0.7 (0.6, 0.8)	0.4 (0.3, 0.5)
White meat	0.3 (0.2, 0.3)	0.3 (0.2, 0.4)	0.4 (0.3, 0.5)	0.3 (0.2, 0.3)	0.3 (0.3, 0.4)	0.3 (0.3, 0.4)	0.1 (0.1, 0.2)	0.3 (0.2, 0.3)	0.6 (0.5, 0.7)	0.3 (0.3, 0.4)	0.3 (0.3, 0.4)

Fish	0.4 (0.3, 0.5)	0.3 (0.2, 0.4)	0.3 (0.2, 0.4)	0.2 (0.2, 0.3)	0.3 (0.2, 0.4)	0.5 (0.4, 0.6)	0.2 (0.1, 0.2)	0.2 (0.2, 0.3)	0.7 (0.5, 0.8)	0.3 (0.2, 0.3)	0.5 (0.4, 0.6)
Fruit, vegetable or vegetable-fruit juices	0.2 (0.1, 0.2)	0.3 (0.2, 0.4)	0.4 (0.3, 0.5)	0.3 (0.2, 0.4)	0.4 (0.3, 0.4)	0.2 (0.2, 0.3)	0.1 (0.1, 0.2)	0.3 (0.2, 0.4)	0.5 (0.4, 0.6)	0.3 (0.3, 0.4)	0.3 (0.2, 0.3)
Sweetened beverages	0.0 (0.0, 0.0)	0.1 (0.0, 0.1)	0.2 (0.2, 0.3)	0.2 (0.1, 0.3)	0.1 (0.1, 0.1)	0.0 (0.0, 0.0)	0.0 (0.0, 0.1)	0.1 (0.0, 0.1)	0.2 (0.1, 0.3)	0.1 (0.1, 0.2)	0.1 (0.0, 0.1)
Alcoholic drinks	0.2 (0.1, 0.3)	0.2 (0.2, 0.2)	0.3 (0.2, 0.3)	0.2 (0.2, 0.3)	0.2 (0.2, 0.3)	0.3 (0.2, 0.3)	0.1 (0.1, 0.1)	0.2 (0.1, 0.2)	0.4 (0.3, 0.5)	0.2 (0.2, 0.3)	0.2 (0.2, 0.3)
Salty snacks	0.0 (0.0, 0.0)	0.1 (0.1, 0.1)	0.4 (0.3, 0.4)	0.2 (0.1, 0.2)	0.2 (0.2, 0.3)	0.1 (0.1, 0.2)	0.2 (0.1, 0.2)	0.1 (0.1, 0.2)	0.2 (0.2, 0.3)	0.2 (0.1, 0.2)	0.2 (0.1, 0.2)
Total fat intake (g)^	2.1 (1.6, 2.7)	2.7 (2.0, 3.4)	5.5 (4.5, 6.5)	3.8 (2.9, 4.6)	3.1 (2.4, 3.8)	3.5 (2.7, 4.2)	1.4 (1.0, 1.8)	3.7 (2.9, 4.5)	5.3 (4.4, 6.2)	3.8 (3.2, 4.4)	2.7 (2.1, 3.3)
Regular fat intake (g)&	2.0 (1.5, 2.6)	2.4 (1.8, 3.0)	5.0 (4.1, 5.9)	3.2 (2.4, 3.9)	2.9 (2.3, 3.6)	3.4 (2.6, 4.1)	1.4 (1.0, 1.8)	3.4 (2.6, 4.2)	4.7 (3.9, 5.5)	3.4 (2.8, 3.9)	2.6 (2.0, 3.2)
Percentage energy from dietary fat	34.0 (33.2, 34.8)	34.3 (33.5, 35.1)	37.9 (36.8, 39.0)	36.0 (35.1, 36.9)	35.0 (34.2, 35.9)	35.2 (34.1, 36.2)	33.2 (32.4, 33.9)	35.2 (34.3, 36.0)	37.9 (36.7, 39.0)	35.9 (35.2, 36.6)	34.3 (33.4, 35.3)

Polish-aMED® – 'Polish-adapted Mediterranean Diet' score (range of points: 0-8), levels (in points): 'lower' (0-4), 'higher' (5-8); the frequency consumption was expressed as a times/day after assigning the values for categories of frequency consumption as follows: 'never or almost never'=0; 'once a month or less'=0.025; 'several times a month'=0.1; 'several times a week'=0.571; 'daily'=1; 'several times a day'=2; ^means as intake of total fat added to food; &means as intake of regular fat added to food, after the reduced-fat margarine used was including; the procedure of the total fat intake, regular fat intake and percentage energy from dietary fat calculation are given in the Materials and Methods section; 95%CI – 95% confidence interval.

Table S4. The sample characteristics by dietary patterns among Polish health care workers (%) or mean (SD).

Variable	Dietary Patterns (tertiles/levels)														
	'Sweet-salty-snack-dairy'				'Lacto-ovo-vegetarian'				'Meat- fats-alcohol-fish'				Polish-aMED®		
	bottom	middle	upper	*p*-Value	bottom	middle	upper	*p*-Value	bottom	middle	upper	*p*-Value	lower	higher	*p*-Value
Sample size (n)	148	148	149		148	149	148		149	147	149		297	148	
Gender															
men	15.5	20.3	24.2	0.1774	21.6	22.1	16.2	0.3684	17.4	15.6	26.8	0.0349	20.9	18.2	0.5131
women	84.5	79.7	75.8		78.4	77.9	83.8		82.6	84.4	73.2		79.1	81.8	
Age (years#)	39.6±11.1	34.0±10.1	32.4±10.2	<0.0001	36.1±12.1	34.7±10.4	35.3±10.1	0.7409	33.5±9.9	36.5±11.1	36.1±11.5	0.0468	35.6±11.4	34.8±9.8	0.9685
<30.0	23.6	47.3	54.4		43.2	45.0	37.2		47.0	38.8	39.6		43.1	39.2	
30.0-39.9	29.1	27.0	26.2	<0.0001	21.6	26.8	33.8	0.2761	28.9	25.9	27.5	0.2812	24.6	33.1	0.2024
40.0-49.9	27.0	15.5	10.1		18.2	16.8	17.6		14.1	22.4	16.1		17.5	17.6	
≥50.0	20.3	10.1	9.4		16.9	11.4	11.5		10.1	12.9	16.8		14.8	10.1	
SES Index#	11.2±1.6	11.6±1.4	11.5±1.3	0.2469	11.2±1.6	11.5±1.4	11.6±1.3	0.1655	11.7±1.2	11.3±1.6	11.2±1.5	0.0186	11.3±1.4	11.6±1.5	0.0243

low	1.4	0.0	0.0		1.4	0.0	0.0		0.0	0.7	0.7		0.3	0.7	
average	30.8	24.5	24.3	0.1733	34.0	23.8	21.8	0.0273	18.4	31.0	30.2	0.0661	30.5	18.5	0.0248
high	67.8	75.5	75.7		64.6	76.2	78.2		81.6	68.3	69.1		69.2	80.8	
BMI (kg/m^{2} #)	24.2±3.3	24.2±2.9	24.3±2.8	0.7958	24.2±3.0	24.1±2.9	24.4±3.2	0.7983	24.0±2.9	24.1±2.9	24.5±3.2	0.3556	24.2±3.0	24.3±3.0	0.8931
underweight (<18.5)	3.4	0.7	0.0		1.4	0.7	2.0		1.3	0.7	2.0		1.3	1.4	
normal weight (18.5-24.9)	53.7	56.8	54.4	0.1787	55.1	56.4	53.4	0.3598	55.7	60.5	48.6	0.3994	54.2	56.5	0.8776
overweight (25.0-29.9)	38.1	39.9	43.0		39.5	42.3	39.2		40.9	34.7	45.3		41.4	38.1	
obesity (≥30)	4.8	2.7	2.7		4.1	0.7	5.4		2.0	4.1	4.1		3.0	4.1	
Chronic diseases	47.2	44.9	22.8	<0.0001	36.1	40.1	38.4	0.7704	32.4	45.1	37.2	0.0784	36.4	41.8	0.2735
Taking medication > 1 year	39.2	37.8	17.4	<0.0001	33.8	30.9	29.7	0.7408	27.5	33.3	33.6	0.4454	30.3	33.8	0.4563
Physical activity[1]															
GLTEQ (summary points#)	37.7±26.9	38.5±29.1	33.2±29.0	0.1614	33.5±32.3	33.9±23.8	41.7±27.7	0.0155	39.8±30.6	36.0±28.0	33.5±26.7	0.3710	34.4±27.5	41.1±30.3	0.0723
GLTEQ (strenuous and moderate points#)	28.7±23.7	29.1±23.9	25.7±25.6	0.2214	26.1±26.6	24.3±19.9	32.7±25.4	0.0204	30.8±26.1	27.2±23.9	25.5±23.1	0.2961	26.6±23.9	30.6±25.6	0.1961
insufficiently active	24.7	26.9	38.3		38.5	28.4	23.8		25.3	31.9	33.6		30.8	29.2	
moderately active	22.5	19.2	19.6	0.2263	17.3	26.3	17.8	0.0656	21.2	17.6	21.8	0.6097	22.7	14.6	0.1925
active	52.8	53.8	42.1		44.2	45.3	58.4		53.5	50.5	44.5		46.4	56.2	
Alcohol drinking															
within the 12 last months	35.4	30.4	40.3	0.2060	34.5	38.3	33.3	0.6495	33.1	27.2	45.6	0.0032	36.4	33.3	0.5297
within the last 10 years	76.2	68.7	80.5	0.0587	77.6	74.5	73.5	0.7010	75.5	71.4	78.5	0.3662	74.0	77.6	0.4135
Smoking status (smoker[2])	54.1	50.0	60.1	0.2113	55.4	57.0	51.7	0.6394	51.7	52.1	60.4	0.2326	53.9	56.5	0.6058
Current smoker	14.9	12.3	22.1	0.0612	23.8	16.1	9.5	0.0043	13.6	15.0	20.8	0.2067	18.9	11.6	0.0495
Vitamin/mineral supplements use[3]	68.9	73.0	70.5	0.7412	63.5	74.5	74.3	0.0586	71.1	76.2	65.1	0.1100	68.4	75.7	0.1094
Number of meals															
1-2	6.1	7.4	11.4		10.8	8.7	5.4		9.4	6.1	9.4		9.8	5.4	
3	27.0	25.7	30.9	0.5118	34.5	27.5	21.6	0.0917	21.5	32.7	29.5	0.3839	33.0	17.6	0.0006
4	48.0	45.9	38.9		37.8	44.3	50.7		47.7	44.2	40.9		40.7	51.4	
≥5	18.9	20.9	18.8		16.9	19.5	22.3		21.5	17.0	20.1		16.5	25.7	
Special diet or intake restrictions	55.4	44.6	22.3	<0.0001	29.1	42.6	50.7	0.0007	49.3	45.6	27.5	0.0002	34.1	54.1	<0.0001
Overall decrease in food consumption	84.5	78.4	61.1	<0.0001	70.9	73.2	79.7	0.1956	83.9	71.4	68.5	0.0051	73.4	77.0	0.4077

Restriction in consumption of:															
dairy	49.0	25.7	12.8	<0.0001	24.3	32.9	29.9	0.2563	26.8	36.1	24.3	0.0654	24.7	37.8	0.0039
fish	19.0	19.0	12.8	0.2482	19.7	17.4	13.6	0.3675	25.5	15.6	9.5	0.0011	18.3	14.2	0.2759
fruits	13.5	8.8	6.7	0.1284	12.2	8.1	8.8	0.4470	6.0	11.6	11.5	0.1822	9.4	10.2	0.7946
raw vegetables	5.4	6.1	4.0	0.7158	8.8	3.4	3.4	0.0531	8.1	4.1	3.4	0.1463	5.7	4.1	0.4625
fats	61.2	56.8	44.6	0.0122	39.9	59.1	63.7	<0.0001	65.8	55.5	41.2	0.0001	50.2	62.3	0.0158
foods in high fat content	77.0	69.6	46.3	<0.0001	53.4	66.4	73.0	0.0016	72.5	70.7	49.7	<0.0001	58.2	76.4	0.0002
sugar and sweets	85.8	74.1	52.0	<0.0001	58.1	73.2	80.8	<0.0001	81.1	74.0	57.0	<0.0001	68.7	74.7	0.1945
potatoes and cereals	41.2	28.4	20.1	0.0003	23.6	28.2	37.8	0.0245	34.9	29.3	25.5	0.2038	27.3	35.1	0.0878
meat and meat products	51.4	45.9	31.5	0.0017	29.1	43.0	56.8	<0.0001	61.7	44.2	22.8	<0.0001	34.7	59.5	<0.0001

SES—socioeconomic status calculated on the basis of place of residence, education level, and self-declared situation of household (description in the Materials and Methods section); BMI—body mass index; [1]data for n=296; [2]ever-smoker (current and/or former smoker); [3]self-declared use of vitamin and/or mineral supplements within the last 12 months; %—sample percentage; [#]mean and standard deviation (SD); *p*-value—level of significance verified with chi^2 test (categorical variables) or Kruskal-Wallis' test (continuous variables); $p < 0.05$—statistically significant.

Table S5. The mean (SD) of the food consumption by mode of the work among Polish health care workers.

Variables	Total sample	Mode of the work		p-Value
		shift	daily	
Size (n)	445	193	252	
Frequency of food consumption (times/day)[&]				
Sugar, honey and sweets	1.3 ±1.4	1.4±1.4	1.2±1.4	0.0088
Milk and milk beverages – natural and cheese curds	1.0 ±0.8	1.0±0.8	1.0±0.9	0.9711
Milk beverages – sweetened and flavoured cheese curds	0.3 ±0.5	0.3±0.5	0.2±0.5	0.0004
Cheese	0.4 ±0.5	0.4±0.5	0.4±0.5	0.7917
Eggs and egg dishes	0.5 ±0.5	0.5±0.5	0.5±0.4	0.8388
Whole grains	0.9 ±0.8	0.8±0.8	0.9±0.8	0.2013
Refined grains	0.6 ±0.6	0.7±0.7	0.6±0.6	0.1298
Breakfast cereals	0.2 ±0.3	0.2 ±0.3	0.2 ±0.3	0.1372
Vegetable based oil	0.4 ±0.4	0.4±0.3	0.5±0.4	0.1757
Animal fats	0.7 ±0.7	0.8±0.8	0.6±0.7	0.0036
Other fats	0.3 ±0.4	0.4±0.5	0.2±0.4	0.0006
Fruits	0.8 ±0.6	0.8±0.6	0.7±0.5	0.9819
Vegetables	0.9 ±0.6	0.8±0.6	1.0±0.7	0.0176
Legumes	0.5 ±0.6	0.5±0.7	0.5±0.6	0.6437
Potatoes	0.4 ±0.4	0.4±0.4	0.3±0.4	0.0045
Nuts and seeds	0.7 ±0.8	0.7±0.8	0.7±0.9	0.1491
Processed meats	0.6 ±0.7	0.8±0.8	0.5±0.6	0.0001
White meat	0.3 ±0.4	0.4±0.4	0.3±0.3	0.0001
Fish	0.4 ±0.5	0.4±0.6	0.3±0.4	0.1592
Fruit, vegetable or vegetable-fruit juices	0.3 ±0.5	0.3±0.5	0.3±0.5	0.1389
Sweetened beverages	0.1 ±0.3	0.1±0.3	0.1±0.3	0.0001
Alcoholic drinks	0.2 ±0.4	0.2 ±0.4	0.2 ±0.4	0.7279
Salty snacks	0.2 ±0.3	0.2 ±0.3	0.2 ±0.3	0.0051
Food consumption (g)[$]				
Cold cereal	9.7±14.0	9.1±13.7	10.2±14.2	0.7129
Skim milk, on cereal or to drink	27.8±69.6	27.6±69.5	27.9±69.8	0.0191
Orange juice or grapefruit juice	42.4±83.7	46.0±89.4	39.6±79.0	0.0143
Fruit (not juices)	74.9±66.6	73.4±68.7	76.0±65.0	0.3254
Eggs, fried or scrambled in margarine, butter or oil	22.7±24.2	21.5±21.7	23.5±26.0	0.9682
Sausage or bacon, regular-fat	3.3±5.5	3.8±6.1	2.9±4.9	0.0005
Beef or pork hot dogs, regular fat	2.7±8.1	3.7±10.4	1.9±5.8	0.0004
Cheese or cheese spread, regular-fat	7.1±9.3	7.2±8.9	7.0±9.5	0.0859
French fries, home fries, or hash brown potatoes	6.8±12.5	7.7±12.5	6.1±12.4	0.0001
Rice	40.0±46.8	40.9±46.4	39.4±47.1	0.3699
Mayonnaise, regular-fat	13.8±10.3	15.7±10.4	12.4±9.9	0.0001
Salad dressings, regular-fat	2.1±4.5	2.5±4.8	1.8±4.2	0.0011
Margarine or butter on bread, rolls, pancakes	2.5±3.6	2.8±3.9	2.3±3.3	0.0781
Margarine or butter on vegetables, including potatoes	0.6±1.3	0.7±1.5	0.4±1.1	0.0072
Margarine, butter, or oil on rice or pasta	0.4±1.0	0.5±1.3	0.2±0.8	0.0012

[&]data from the 62-item FFQ-6®, the frequency consumption was expressed as a times/day after assigning the values for categories of frequency consumption as follows: 'never or almost never'=0; 'once a month or less'=0.025; 'several times a month'=0.1; 'several times a week'=0.571; 'daily'=1; 'several times a day'=2; [$]data from the Quick Food Scan of the National Cancer Institute; $p < 0.05$—statistically significant.

Table S6. Fat intake, food consumption, and dietary patterns in association with the percentage of energy from dietary fat among Polish health care workers (% or mean (SD).

Variable	Percentage energy from dietary fat		*p*-Value
	20-35%	>35%	
Sample Size	257	188	
Total fat intake (g)[#] [^]	1.5±2.6	6.0±5.8	<0.0001
Regular fat intake (g)[#] [&]	1.4±2.3	5.5±5.5	<0.0001
% energy from dietary fat[#]	31.7±2.1	40.4±5.7	<0.0001
Food consumption (g) [#] [$]			
Cold cereal	12.3±15.3	6.1±11.0	0.0091
Skim milk, on cereal or to drink	37.6±85.0	14.2±35.9	0.8714
Orange juice or grapefruit juice	38.0±89.1	48.4±75.3	<0.0001
Fruit (not juices)	79.5±72.5	68.7±57.0	0.5565
Eggs, fried or scrambled in margarine, butter or oil	16.4±20.2	31.2±26.5	<0.0001
Sausage or bacon, regular-fat	1.3±2.3	6.0±7.2	<0.0001
Beef or pork hot dogs, regular fat	0.7±1.3	5.4±11.9	<0.0001
Cheese or cheese spread, regular-fat	5.2±8.2	9.7±10.0	<0.0001
French fries, home fries, or hash brown potatoes	3.7±8.4	11.1±15.5	<0.0001
Rice	30.3±42.4	53.3±49.2	<0.0001
Mayonnaise, regular-fat	8.2±4.2	21.5±11.1	<0.0001
Salad dressings, regular-fat	0.7±1.3	4.0±6.3	<0.0001
Margarine or butter on bread, rolls, pancakes	1.2±2.2	4.3±4.3	<0.0001
Margarine or butter on vegetables, including potatoes	0.2±0.6	1.1±1.8	<0.0001
Margarine, butter, or oil on rice or pasta	0.1±0.4	0.7±1.5	<0.0001
Frequency of reduced-fat margarine use			
didn’t use/almost never	93.3	83.3	
about 1/4 of the time	5.1	13.4	
about 1/2 of the time	0.0	0.0	0.0019
about 3/4 of the time	0.8	0.0	
almost always or always	0.8	3.2	
Fat content in the diet (self-declared)			
low	18.9	8.7	
medium	68.9	66.8	0.0002
high	12.2	24.5	
Consistent assessment of comparison of fat content in the diet (objective vs subjective)	21.3	24.5	0.4298
Dietary Patterns (tertiles/levels)			
'Sweet-salty-snack-dairy'			
bottom	40.5	23.4	
middle	37.7	27.1	<0.0001
upper	21.8	49.5	
‘Lacto-ovo-vegetarian’			
bottom	31.5	35.6	
middle	33.9	33.0	0.6302
upper	34.6	31.4	
‘Meat- fats-alcohol-fish’			
bottom	44.4	18.6	
middle	33.1	33.0	<0.0001
upper	22.6	48.4	
Polish-aMED® score (points)[#]	4.1±1.7	3.6±1.7	0.0129
lower (0-4 points)	63.0	71.8	
higher (5-8 points)	37.0	28.2	0.0523

Polish-aMED® – Polish-adapted Mediterranean Diet (range of points: 0-8); ^means as intake of total fat added to food; [&]means as intake of regular fat added to food, after the reduced-fat margarine used was including; the procedure of the total fat intake, regular fat intake and percentage energy from dietary fat calculation are given in the Materials and Methods section; [$]data from the Quick Food Scan of the National Cancer Institute; % – sample percentage; [#]mean (SD); p-value – level of significance assessed by chi^2 test (categorical variables) or Kruskal-Wallis’ test (continuous variables); $p < 0.05$—statistically significant.
